# Neuro-affirmative support for autism, the Double Empathy Problem and monotropism

**DOI:** 10.3389/fpsyt.2025.1538875

**Published:** 2025-03-13

**Authors:** Mark Brosnan, Louis John Camilleri

**Affiliations:** ^1^ Centre for Applied Autism Research (CAAR), University of Bath, Bath, United Kingdom; ^2^ Department for Inclusion and Access to Learning, University of Malta, Msida, Malta

**Keywords:** autism, neuro-affirmative support, digital social narratives, double empathy problem, monotropism

## Abstract

Conceptualising autism within a neurodiversity approach raises fundamental questions regarding the nature of the goals pursued in autism support and who is responsible for achieving these goals. The Double Empathy Problem considers deficits in social communication as residing between autistic and non-autistic communicators, rather than solely within the autistic individual. This is important as autistic individuals can have different perceptions of what appropriate goals for autism support should be, when compared to (non-autistic) family, friends and professionals. Monotropism highlights the importance of engaging with the interests of the autistic individual when considering support. This perspective considers the extent to which autistic individuals can self-set and self-achieve autism support goals? Social narratives have a specific goal and explicit description of how to achieve this goal and what the outcome of achieving the goal will be. The Stories Online For Autism app (SOFA-app.com) develops and delivers social narratives for autistic individuals. The SOFA-app has proven to be highly acceptable and effective in supporting autistic individuals. Initially our research focussed on family, friends and professionals developing autism support for autistic children. Subsequently we extended this methodology to explore the self-set goals of autistic adults and children as well as capacity to self-achieve these goals successfully. Digital support for the development and delivery of social narratives to support self-set goals for autistic individuals is recommended. Addressing the Double Empathy Problem and supporting self-set goals are also considered alongside the implications of preferences associated with Monotropism to argue this approach can be considered neuro-affirmative.

‘*Until recently, nearly no research or implementation work has sought the input of autistic people in regard to the design of interventions and, more importantly, how the goals for such interventions are prioritised and determined*’ (1).

## Neuro-affirmative approaches, the Double Empathy Problem and monotropism

Clinically, autism is defined as a condition characterised by deficits in social communication and patterns of restricted and repetitive behaviour (2, 3). This clinical characterisation of autism has been challenged by neurodiversity approaches that consider disability as emerging from an interaction of individual and context, allowing interventions to either change individuals in limited ways (e.g., teaching skills, using medication to manage difficulties) or to change environments and societies (4; see also 5–7). The neurodiversity approach also challenges the acceptability of interventions focused on the ‘normalisation’ of autistic behaviours (e.g., echolalia, perseveration, and stereotypy), an intervention focus which can result in diminished mental health of autistic individuals (8–10). Conceptualising autism within a neurodiversity approach aligns with considering deficits in social communication as residing between autistic and non-autistic communicators, rather than solely within the autistic individual. This ‘disjuncture in reciprocity between two differently disposed social actors’ has been called the Double Empathy Problem (DEP: 11–14, see also 15). The DEP is evidenced by effective communication between two autistic communication partners and between two non-autistic communication partners (i.e. both within-neurotype), as well as communication breakdown between an autistic and non-autistic communication partner (i.e. cross-neurotype: 16–22).

Autism has also been characterised as being monotropic, that is a single-minded cognitive style, focussed on attending to things that capture interest (and challenges shifting attention to things not of interest; 23; see also 24). According to the monotropism theory of autism, autistic attention is characterised by hyper-focus and intrinsically rewarding ‘flow states’ (25). Murray et al. (23: 143) argue that monotropism results in autistic preferences for ‘a narrow range of predictable attractors, and learning and thinking strategies which do not depend on simultaneous arousal of a number of distinct interests, such as comparisons, metaphors, contextualization and social motivation’. From this perspective, neuro-affirmative support entails joining the autistic individual in their attention tunnel rather than trying to get them to come out (26). Murray (26) argues that this ‘must include a learning environment in which they are motivated to learn through their own explorations, in which the structure facilitates learning for them rather in obstructing it’. Thus, neuro-affirmative support should align with the interests of the autistic individual - whether social or non-social.

## Neuro-affirmative support for autism

The DEP and monotropism are therefore crucial for understanding appropriate support for members of the autistic community as existing programmes of support dominantly represent a pathological, outsider view of autism (27). A neurodiversity approach informing support for autism (‘neuro-affirmative’) is an urgent priority, and would ideally be neurodivergent-led putting lived experiences ‘centre stage’ (1, 8, 27–30). From the neuro-affirmative approach, traditional programmes of support focused on reducing autistic ‘symptoms’ can exacerbate mental health issues, as autistic mental health is positively correlated with autism acceptance and perceived quality of support provided, not necessarily with lack of ‘symptomatologic severity’. Therefore, the development and dissemination of neuro-affirmative support is key for addressing the autism mental health crisis (31; see 32 for review). When conceptualising neuro-affirmative support for autism, Chapman and Bovell (33) propose three key considerations: 1) conceptualising autism within the neurodiversity approach; 2) the goals pursued in autism support; and 3) who is responsible for achieving these goals (see also 27, 34).

## Goals for neuro-affirmative support

Recent research has explored perspectives on who should have a leading role in setting the goals for supporting autistic individuals and what these goals should be. Dwyer et al. (35) compared the views of members of the autistic community with views from the non-autistic broader autism community (family, friends, professionals). When compared to the non-autistic group, the autistic group much less often endorsed parents and trained professionals having leading roles in setting intervention goals, and more often supported involvement of autistic consultants in developing goals (35). This is important as autistic people can have different priorities for intervention goals than non-autistic members of the broader autism community. Both autistic and non-autistic groups concurred on the importance of well-being intervention goals for autism. In addition, however, the non-autistic group endorsed ‘normalisation’ and ‘adaptive skills’ intervention goals significantly more than the autistic group (large and medium effect sizes respectively). The autistic group, on the other hand, endorsed intervention goals of ‘supportive environments’ and ‘societal reform’ more than the non-autistic group (small effect: 36). Taken together, the neurodiversity approach denounces normalisation, but is open to individualised supports with strong support for autistic leadership (especially among autistic people) and for societal reform (35).

Thus, autistic individuals can have different perceptions of what appropriate goals for support should be, when compared to family, friends and professionals (see also 37, 38). Schuck et al. (39) conducted a survey of autistic adults to identify common themes in their perceptions of appropriate intervention goals. The authors highlighted several areas where professionals could help autistic individuals obtain a higher quality of life, namely: understanding and emphasizing an autistic way of being (addressing the DEP), encouraging autonomy and self-advocacy, using appropriate intervention procedures that do not cause disproportionate psychological or physical harm, and looking beyond outward behaviour to help solve root causes (see also Baiden et al. (40)). Whilst many autistic individuals can set their own goals, when developing support autistic involvement in goal-setting is the exception due to time and resource limitations in supporting the development and implementation of (autonomous) self-set goals (41, 42). Despite these discrepancies, there is high acceptability for supporting self-set goals in autistic individuals, from autistic individuals themselves, their family, friends and professionals (37). Autistic individuals need individualised support to meet their self-determined needs, however, few evidence-based interventions have been developed to provide this support for autistic individuals (43).

## Recommendation: digital support for social narratives

One evidence-based support for autistic individuals to self-set and self-deliver support for their own intervention goals is the Stories Online For Autism app (SOFA-app.org), which we co-developed with the autistic community (44). Initially the SOFA-app was found to effectively support family, friends and professionals to set goals for autistic individuals, develop plans for autistic individuals to attain these goals, and track their progress towards these goals in community-based settings (45–50). Recently, we have also found the SOFA-app to be effective for autistic individuals to self-set goals, develop plans to attain these goals, and self-monitor progress towards these goals (51, 52). The SOFA-app combines goal-based outcomes methodology with social narratives. Social narratives, such a Social Stories or comic strip methodologies, have a specific goal and explicit description of how to achieve this goal and what the outcome of achieving the goal will be. The aim of this support is to provide an accurate understanding of social situations, given that the social capacity of autistic individuals is presumed to stem from a lack of access to social information and not necessarily a lack of ability to understand and respond to that information (53). Autistic authors have argued this approach can be considered neuro-affirmative when ‘provision of equalizing information that allows autistic individuals to act from a basis of more complete understanding of the social expectations in the contexts they find themselves is a respectful way to equip autistic individuals for navigation of a society that was not built for us’ (54: 327). Social narratives have been found to be highly effective for increasing desirable behaviours, decreasing undesirable behaviours, enhancing skill development and supporting transitions for autistic individuals (see 55 for review).

## Recommendation: support SMART goals

Within the SOFA-app, autistic individuals use the SMART (Specific, Measurable, Achievable, Relevant, Time-bound) goal framework to identify a goal they would like to achieve, that is rigorous enough to be the focus of support (56, 57). The SOFA-app then supports the development of an appropriately structured social narrative to achieve this goal. The social narrative is then accessed (i.e. read via the app) daily and closeness-to-goal monitored daily, for a period of one week (58). Camilleri et al. (52) found that autistic adults could use the SOFA-app to effectively identify SMART goals, develop social narratives to attain these goals, and self-monitor their closeness-to-goal. The intervention was also found to be highly acceptable for autistic adults. Camilleri et al. (51) extended this to autistic children and found that older children (secondary school, aged 11+) could also effectively use the SOFA-app in this manner. Younger autistic children (primary school, aged 5+) also used the SOFA-app effectively, though needed parental guidance in identifying SMART goals, social narrative development and reminders to access the social narrative daily. For all aged groups, autistic individuals successfully achieved their self-set SMART goals.

## Recommendation: digital support for non-social narratives

When asking autistic adults what self-set goals they would like to achieve, many identified non-social goals of gaining a skill or completing a task (52). The SOFA-app was able to effectively support both self-set social and non-social goals. Qualitative analysis suggested that autistic adults felt the SOFA-app supported reflection on the self-set goal, the breaking down of the process of achieving the goal into meaningful parts, the creation of rules to achieve the goals, and the formation of predictions about achieving the goals. Camilleri et al. (52) suggest that the SOFA-app may therefore support differences in executive functioning and relative rule-based strengths associated with autism (59). It was argued above that when conceptualising neuro-affirmative support for autism, key considerations are the goals pursued and who is responsible for achieving these goals (see 27, 33, 34). When autistic adults are responsible for achieving their own support goals, neuro-affirmative support needs to be able to address non-social (as well as social) goals.

## How can the SOFA-app mitigate the Double Empathy Problem?

Autistic users of the SOFA-app report a sense of autonomy when addressing self-set goals (52), and the SOFA-app could therefore be considered a neuro-affirmative support for autism, addressing the self-set intervention goals of the autistic community. However, Schuck et al. (39) report reflections on appropriate intervention goals proposed by the autistic community: ‘Why is the autistic person the only one that needs to be flexible? Helping both autistic and non-autistic people understand how others experience empathy and listening differently and using that understanding as a lens to be more accepting and supportive improves communication skills for everyone’ as well as ‘Allowing the autistic person to drive that [increasing independence] while being there to offer support in helping them to make those decisions (including helping them to make better informed decisions) and helping them to have more tools/options to choose from’. Thus, in addition to supporting autonomy, the extent to which this approach can support independence needs to be evidenced. The reflections above from the autistic community highlight how independence may involve non-autistic people better understanding and supporting the autistic experience.

This raises the question of whether the SOFA-app could address the disjuncture in reciprocity between autistic and non-autistic people to address the Double Empathy Problem (59). Clearly the DEP is a multifaceted and if this approach can support autistic people achieving self-set goals, develop a focus on societal reform by supporting acceptance (including non-autistic groups), and enable autistic and non-autistic individuals to co-develop interdependent support, there is potential for mitigating the DEP and supporting a ‘Double Empathy Solution’. The SOFA-app has the potential to support the mitigation of the DEP by facilitating the co-creation of both social and non-social narratives. Through this collaborative narrative process, the SOFA-app fosters interdependent support, encouraging mutual understanding and shared meaning. By guiding users toward the development of a singular, goal-oriented narrative, the SOFA-app creates a structured and engaging context where both individuals can align their focus. This process not only promotes deep engagement—a characteristic strength of monotropic attention—but also provides a common framework that facilitates reciprocal communication.

Furthermore, the narrative co-creation approach encourages both parties to adapt their perspectives, reducing misalignment in communication styles and enhancing relational attunement. By offering a structured yet flexible digital platform, the SOFA-app can serve as a bridge, fostering shared cognitive spaces where autistic and non-autistic individuals can engage in meaningful, goal-directed collaboration. Additionally, in collaborative narrative or co-creation contexts, the shared goal of developing a structured narrative can foster interpersonal connection without necessitating abrupt shifts in focus. This makes narrative-based tasks particularly effective in promoting meaningful social engagement between autistic and non-autistic individuals, as both parties work within a defined structure that accommodates their cognitive styles.

## How can the SOFA-app support autistic individuals with a highly focused and narrow range of interests (monotropism)?

Narrative development/creation can serve as a highly structured and immersive task that supports deep, sustained focus, making it particularly well-suited for individuals with a monotropic cognitive style. This process leverages structure, and intrinsic motivation, offering a supportive framework that enhances engagement and possibly also minimises cognitive overload. For monotropic individuals, shifting attention between multiple tasks or topics can be challenging and mentally exhausting. The structured nature of narrative development provides a single, coherent focal point, allowing the channelling of cognitive resources into a sustained and meaningful activity. By embedding schedules, routines, and visual supports, the process becomes more accessible and manageable, reducing the cognitive demands associated with unexpected changes or competing distractions.

Monotropic attention thrives in environments where predictability and consistency are maintained. The act of developing a narrative introduces a clear sequence of events with a defined goal. This structured progression provides a cognitive anchor. Thus, the process of constructing a coherent and purposeful narrative aligns with the natural tendencies of monotropic individuals to engage deeply with specific interests. Thus, for individuals with a monotropic cognitive style, narrative creation can be viewed as not merely a creative exercise but a strategic, structured approach that leverages deep focus as a strength. The SOFA-app may therefore provide an appropriate digital environment to support monotropic individuals. The SOFA-app facilitates self-set goals through breaking down the process of achieving the goal into meaningful parts, the creation of rules to achieve the goals, and the formation of predictions about achieving the goals (see 52, see above), that align with the autistic preferences described by Murray (23, see above).

## In conclusion

Considering neuro-affirmative support for autism may therefore be consistent with perspectives raised by monotropism and the DEP. This Perspective article proposes that the SOFA-app can be considered neuro-affirmative support for autism. A clear limitation is a lack of empirical support currently exploring whether the DEP or monotropism are supported by the SOFA-app, and this Perspective article proposes an agenda to address this gap in the literature. Having been co-developed with the autistic community, the SOFA-app is free (no costs at all) to download at SOFA-app.org.

## Data Availability

The original contributions presented in the study are included in the article/supplementary material. Further inquiries can be directed to the corresponding author.
